# Endovascular repair with extracorporeal membrane oxygenation as a rescue strategy for aortobronchial fistula: a case report

**DOI:** 10.1186/s40981-017-0103-8

**Published:** 2017-06-09

**Authors:** Kyosuke Takahashi, Misa Kajitani, Takaaki Kamada, Wataru Takayama, Yoshiro Kobayashi

**Affiliations:** 1 Department of Anesthesia and Critical Care, Jichi Medical University Saitama Medical Center, 1-847 Amanumacho, Omiya-ku, Saitama city, Saitama 330-8503 Japan; 2 Department of Anesthesia, Kawasaki Saiwai Hospital, 31-27 Omiyacho, Saiwai-ku, Kawasaki city, Kanagawa 212-0014 Japan; 3 Department of Anesthesia, National Hospital Organization Tokyo Medical Center, 2-5-1 Higashigaoka, Meguro-ku, Tokyo, 152-8902 Japan; 4 Department of Anesthesia and Critical Care, 1-847 Amanumacho, Omiya-ku, Saitama city, 330-8503 Japan

**Keywords:** Endovascular repair, Extracorporeal membrane oxygenation, Aortobronchial fistula, Thoracic aortic aneurysm, Massive hemoptysis

## Abstract

Aortobronchial fistula (ABF) is a rare and potentially lethal complication of thoracic aortic replacement surgery. Currently, thoracic endovascular aortic repair (TEVAR) has emerged as a less invasive alternative to open surgery for ABF to facilitate prompt hemostasis. However, there are no published reports of TEVAR for ABF, particularly for presentation with life-threatening respiratory failure from massive hemoptysis.

A 48-year-old male patient, who had recently undergone aortic root and arch replacement due to aortic dissection, was transferred to the emergency department with massive hemoptysis and severe dyspnea. A single-lumen endotracheal tube was immediately placed in the right main bronchus to protect the nonbleeding lung from spillage of blood. Chest computed tomography (CT) showed leakage of contrast material from the distal anastomosis of the aortic graft and consolidated lung tissue adjacent to the leakage. He was diagnosed with an ABF following aortic arch replacement, and an emergency TEVAR was performed. After adequate hemostasis, severe hypercapnia remained uncorrected despite the maximum ventilatory support. Thus, venovenous extracorporeal membrane oxygenation (VV ECMO) was immediately initiated, and severe respiratory acidosis improved dramatically. Furthermore, VV ECMO facilitated prompt bronchoscopic washout of the remaining blood clot without any danger of respiratory collapse and was weaned off successfully after 5 days as ventilation improved.

This case demonstrates that emergency TEVAR in combination with VV ECMO can be a rescue strategy for massive hemoptysis from an ABF.

## Background

Aortobronchial fistula (ABF) is a rare and potentially lethal complication of thoracic aortic replacement surgery [1]. Thoracic endovascular aortic repair (TEVAR) is a less invasive alternative to open surgery for ABF to facilitate prompt hemostasis [2]. However, there are no published reports of TEVAR for ABF, particularly for presentation with life-threatening respiratory failure from massive hemoptysis. We describe a successful combination of venovenous extracorporeal membrane oxygenation (VV ECMO) with emergency TEVAR for a patient with ABF and life-threatening hemoptysis.

## Case presentation

**Fig. 1:**
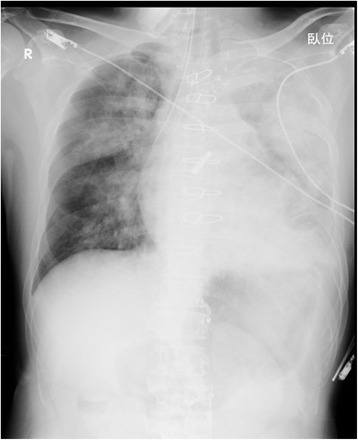
The chest X-ray shows diffuse consolidation of the left lung due to massive bleeding. A single-lumen endotracheal tube is placed in the right main bronchus

The patient was a 48-year-old male, who had undergone aortic root and arch replacement due to aortic dissection 10 months prior to the admission. He had cough and hemoptysis 1 month prior to the admission, but he had not come to the hospital. He was transferred to the emergency department with massive hemoptysis and severe dyspnea. Even after initial resuscitation of hemorrhagic shock, respiratory failure continued because of uncontrolled massive hemoptysis; thus, a single-lumen endotracheal tube was immediately placed in the right main bronchus to protect the nonbleeding lung from blood spillage. Arterial blood gas analysis showed severe respiratory acidosis (pH, 7.13; pCO_2_ 76.2 mmHg, pO_2_ 92.8 mmHg; HCO_3_^−^ 25.2 mEq/L; BE −4.0 mmol/L). Chest computed tomography showed leakage of contrast material from the distal anastomosis of the aortic arch, and chest X-ray showed consolidated lung tissue adjacent to the leakage (Fig. 1). He was diagnosed with ABF following aortic arch replacement, and emergency TEVAR was scheduled. At this time, massive hemothorax and lung bleeding from the ABF had led to not only potentially lethal hypercapnic respiratory failure but also hemodynamic instability. Therefore, endovascular treatment was preferred to achieve hemostasis and avoid prolonged operative time, because extensive pleural adhesions were anticipated from a history of repeat thoracotomy.

Emergency TEVAR under general anesthesia was successfully completed within 30 min and provided both adequate hemostasis and hemodynamic stability. He was transfused with 22 units of packed red blood cells, 20 units of platelet concentrate, and 18 units of fresh frozen plasma until adequate hemostasis was achieved. Despite the maximum manual ventilatory support and administration of tromethamine, severe hypercapnia and life-threatening acidosis remained uncorrected. Worse still, peak airway pressure higher than 60 mmH_2_O was required to achieve tidal volume of 5 ml/kg. Arterial blood gas analysis showed the following: pH, 6.88; pCO_2_, 199.9 mmHg; pO_2_, 129.0 mmHg; HCO_3_^−^, 37.3 mEq/L; and BE, 3.3 mmol/L. Thus, VV ECMO was immediately initiated as a bridge therapy for respiratory recovery. Drainage cannula was inserted from the left femoral vein and placed in the inferior vena cava. Return cannula was inserted into the right internal jugular vein. Intravenous heparin (2000 units) was administered before cannula insertion to maintain activated clotting time (ACT) values lower than 200 s. Additional heparin was not required to maintain adequate anticoagulation during the surgery.

Soon after the initiation of ECMO, the severe respiratory acidosis dramatically improved to a pH of 7.34, a pCO2 of 39.3 mmHg, and a bicarbonate level of 21.0 mEq/L. Sufficient oxygenation was provided without ventilation during maintenance with ECMO; therefore, fiber-optic bronchoscopy was performed to clear the airways of the remaining blood clots without any danger of respiratory collapse. Additionally, endotracheal tube in the right main bronchus was replaced to the trachea in the operation room. Intravenous heparin was continued after ICU admission because there were no signs of hemoptysis. The aim of ACT was 180 to 200 s, and there was no obvious bleeding from the bronchus during ECMO therapy. For the purpose of preventing rebleeding, positive end-expiratory pressure (PEEP) was maintained above 10 cmH_2_0. Propofol and dexmedetomidine were administered to provide adequate sedation. The dose of these drugs were adjusted aiming to achieve a Richmond Agitation Sedation Scale of 0 to −2. Despite the additional blood, components were required for few days to correct for anemia and coagulation disorder, and he was hemodynamically stable without vasopressors. Although hypercapnia was corrected after removing blood clots, subsequent oxygenation failure emerged after the ICU admission impeded weaning from VV ECMO for 5 days. Fluid removal by continuous renal replacement therapy improved oxygenation, and VV ECMO was weaned off uneventfully on postoperative day 5 (see Table 1).

**Table 1:** Ventilator setting and clinical parameters during ECMO therapy

		POD0									
		At the ED	Pre-ECMO	On ECMO	ICU admission	POD1	POD2	POD3	POD4	POD5	POD6 (after weaning off ECMO)
Mechanical ventilation	Mode	–	N/A	A/C (PC)	A/C (PC)	A/C (PC)	A/C (PC)	A/C (PC)	A/C (PC)	A/C (PC)	A/C (PC)
	FiO_2_	–	1	1	0.4	0.6	0.5	0.4	0.4	0.5	0.5
	PEEP (cmH_2_O)	–	N/A	15	10	15	15	15	12	14	12
	Plateau pressure (cmH2O)	–	N/A	30	20	23	23	20	25	26	22
	Frequency (/min)	–	N/A	10	6	6	6	6	10	12	12
	Tidal volume (ml)	–	N/A	100	140	120	150	150	310	420	460
ECMO	FiO_2_	–	–	0.7	0.6	1	0.85	0.6	0.7	0.3	–
	Sweep gas (L/min)	–	–	8	5	3.5	3.5	2.5	3.5	3.5	–
	Pump flow (L/min)	–	–	3	3	2.9	3	3.1	3.1	1.5	–
	Unfractionated heparin (units/h)	–	–	0	260	340	340	250	750	800	500
Atrial blood gas	pH	7.138	6.88	7.345	7.368	7.447	7.494	7,424	7.465	7.419	7.372
	PaCO_2_(mmHg)	76.2	199.9	39.3	38.3	42.5	40	46.1	39.5	42.4	40.3
	PaO_2_(mmHg)	92.8	129	73	86.3	44.7	83.5	83.5	76.9	67.8	86.9
	HCO_3_^−^(mmol/L)	25.2	37.3	21	21.5	28.7	27.5	29.5	27.8	26.8	22.9
	Lactate (mmol/L)	4.5	4.13	N/A	8.57	2.27	1.83	1.56	1.3	1.04	0.78
Coagulation	ACT (s)	–	–	198	239	190	196	187	209	182	183
Water balance	Water balance (ml/day)	4340	–	–	8840	2124	3583	1041	−2410	184	1697

*A/C* (*PC*) assist and control (pressure control), *ACT* activated clotting time, *CMO* corporeal membrane oxygenation, *ECMO* extracorporeal membrane oxygenation, *ED* emergency department, *FIO2* fraction of inspired oxygen, *N/A* not applicable, *PEEP* positive end-expiratory pressure, *POD* postoperative day

However, on postoperative day 12, he developed fever and distributive shock. Contrast CT indicated mesenteric artery embolism and multiple cerebral infarctions. From these findings, intestinal necrosis and sepsis due to bacterial translocation were suspected. Despite the intensive therapy including resection of the necrotic intestine, his infection was difficult to treat and the patient died on postoperative day 56.

### Discussion

Hemoptysis is the first and often only symptom of ABF, a rare complication of thoracic aortic replacement surgery [1]. Once a massive hemoptysis occurs from ABF, patients require urgent medical treatment because of a double risk of death from hypovolemic shock by uncontrolled bleeding and bronchial tree obstruction by blood clots. Therefore, prompt diagnosis and hemostasis are essential to decrease mortality [3, 4]. TEVAR is a less invasive alternative to open surgery for ABF to facilitate prompt hemostysis [2]. Although there are several reports of successful management with VV ECMO in patients with pulmonary hemorrhage or hemoptysis [5–8], to our knowledge, this is the first case of ABF with life-threatening hemoptysis treated successfully under VV ECMO after emergency TEVAR.

Although ECMO was successfully applied in some reported cases of thoracic aortic rupture [9] and lung bleeding [5–8], it remains unclear whether ECMO can be safely applied in massive hemoptysis; in other words, systemic anticoagulation and thrombocytopenia during ECMO are debatable. However, we focused on immediate correction of life-threatening acidosis, consequently leading to recovery of hemostatic function, on the assumption that severe acidosis could probably induce coagulopathy by fibrinogen degeneration [10] and platelet functional impairment [11]. Moreover, it was of utmost importance that ECMO support could allow the patient to undergo prompt bronchoscopic washout of the remaining blood clots from the lower airway without any danger of respiratory collapse. Emergency physicians must be aware of these potential benefits of VV ECMO as a bridge for life-threatening hemoptysis.

Ventilator management for ABF is a clinical challenge. According to the Extracorporeal Life Support Organization (ELSO) guideline [12], a lung rest setting is recommended after initiation of ECMO. Low plateau inspiratory pressure (under 25 cm H_2_O) is desirable and PEEP is usually set between 5–15 cmH_2_O. However, low inspiratory pressure could predispose to bleeding for patients with hemoptysis. Similar to our case, a lung bleeding patient was successfully managed with higher airway pressure (max plateau pressure 35 cmH_2_O for a day) in a previous report [8]. In terms of hemostasis, transient high airway pressure may be permitted until bleeding stops.

A balance between preventing thrombosis (anticoagulation) and bleeding risk has been a difficult clinical dilemma. In our case, heparin was continuously administered during ECMO therapy. Nevertheless, there was an embolic event after weaning of ECMO. In addition to the use of ECMO, aortic dissection could predispose to systemic hypercoagulation and thromboembolism. Although antithrombin III (AT) was not examined, AT deficiency was also suspected because of increasing needs of heparin dose during ECMO therapy. In consideration of avoiding hemorrhage, we aimed ACT below 200 s according to the previous reports [5–7]. However, there was a case of successful VV ECMO for alveolar hemorrhage under a mean ACT of 274 s [8]. Heparin administration aiming for higher ACT levels and/or AT replacement might have prevented the fatal mesenteric artery embolism, provided that there were no signs of bleeding complications. Anticoagulation therapy should be carefully titrated with observing clinical presentations.

On the other hand, the emergency endovascular approach is a promising treatment option for ABF and acute massive hemoptysis, assuming that surgical repair through a left thoracotomy approach may be technically difficult due to extensive pleural adhesions or vascular abnormalities.

## Conclusions

This case demonstrates that emergency TEVAR in combination with VV ECMO can be an attractive rescue strategy for massive hemoptysis in a patient with an ABF.

## Abbreviations

ABF: Aortobronchial fistula
CT: Computed tomography
TEVAR: Thoracic endovascular aortic repair
VV ECMO: Venovenous extracorporeal membrane oxygenation
